# The road to a first-class research institute: creating an exceptional environment, recruiting outstanding scientists, producing high-impact work, and nurturing a culture of innovation—Preface to the 60th anniversary special issue

**DOI:** 10.1007/s41048-018-0069-8

**Published:** 2018-09-14

**Authors:** Rui-Ming Xu

**Affiliations:** Institute of Biophysics, Chinese Academy of Sciences, Beijing, 100101 China

A series of major accomplishments in modern physics during the 1950’s led to the rise of biophysics as a discipline and its development throughout the world, and it was also urgent to fill the gap in China’s biophysics research. In order to support the national defense project of the “Atomic and hydrogen bombs and man-made satellites,” through the efforts of Dr. Shizhang Bei, the State Council of China approved the transformation of the Beijing Institute of Experimental Biology into the Institute of Biophysics (IBP), Chinese Academy of Sciences (CAS) on 26th September, 1958. In the same year, Dr. Bei also founded the Department of Biophysics at the University of Science and Technology of China. China has since seen rapid development in biophysics.

In the 60 years since its establishment, the direction of the Institute of Biophysics has been in line with principles proposed by Dr. Bei: integrate theory with practice, align work with national needs, and strive to reach and surpass global standards. The Institute has attracted a group of outstanding scientists and made many great accomplishments in scientific research, leading and promoting research and development in China on radiobiology, cosmology, cell biology, enzymology, structural biology, membrane biology, neurobiology, biocybernetics, and biophysical engineering. In doing so, it has made an important contribution to the development of the life sciences in China.

In the early years after its establishment, the Institute regarded the completion of state-level projects as an honorable obligation. To meet national needs for the development of atomic and hydrogen bombs and the peaceful usage of atomic energy, the Institute undertook research on important topics such as the long-term effects on animals of nuclear radiation from nuclear weapons testing. This provided a reliable scientific basis for understanding the hazards of nuclear explosions, developing protective measures, and establishing national standards for radiation safety. In cooperation with a national artificial satellite launch and interstellar travel initiative, the Institute undertook the biological experiments associated with the first batch of biological sounding rockets. This work laid a solid foundation for the development of China’s human spaceflight program. The undertaking and completion of state-designated tasks has facilitated the application of new technologies in biophysical research. The Institute has pioneered the successful development of biophysical instruments such as paramagnetic resonance spectrometers, ultracentrifuges, fluorescence spectrophotometers, and automatic liquid scintillation spectrometers. This has not only ensured the completion of state missions and allowed China to compete in related fields, but has also contributed to the development of life science instrumentation in China.

Since China’s Reform and Opening-up, the Institute—under the strong leadership and generous support of the CAS—has set the goal of developing into a world-class institute in the field of the life and health sciences. IBP also aims to play a leading role in innovation within China’s national innovation framework, by implementing the “Knowledge Innovation Program,” the “Innovation 2020” initiatives, the “Pioneer Initiative,” and constructing a CAS Center for Excellence in Biomacromolecules. Focusing on cutting-edge disciplines such as protein science, brain and cognitive science, infection and immunity, and nucleic acid biology, IBP has sought to optimize the arrangement of different disciplines, foster emerging disciplines, and promote interdisciplinary interactions. The Institute has both attracted and grown top talent, built innovative teams, and developed solid research platforms such as State key laboratories and CAS key laboratories, while building and steadily improving support for scientific research platforms, and strengthening innovative research and the development of key equipment in the life sciences. The Institute has significantly improved its capacity for original scientific and technological innovation, and has produced a series of major, original research accomplishments.

The Institute has led or served as the main participating institute in several projects that have been listed among the top 100 major achievements of CAS on its 60th anniversary, include the total synthesis of yeast alanine transfer RNA, quantitative relationship between the modification of protein functional groups and their biological activities, crystal structure of the main light-harvesting complex (LHC-II) of spinach, and crystal structure of porcine insulin. The “Structure–function relationships in eukaryotic membrane proteins and protein complexes” is listed among the top 25 major scientific and technological accomplishments of CAS during China’s 12th Five-Year Plan period. To date, the Institute has received two first prizes and 11 second prizes in the State Natural Science Awards, four second prizes in the State Scientific and Technological Progress Awards, and dozens of major awards granted by CAS and other ministries. IBP’s publication record has led it to being listed among the advanced research organizations internationally. Leveraging its accomplishments in basic research and technology development, the Institute has developed a number of high-tech products, incubated and established several high-tech enterprises such as BioSino Bio-Technology & Science Inc. The Institute also established joint laboratories, bases for tech-transfer, and translational medicine research institutes in collaboration with local governments, institutes, universities, hospitals, and enterprises. These efforts have benefited both society and the economy. In the evaluation during the 12th Five-Year Plan period, the evaluation committee stated that the Institute “is a domestic leader and among the first-tier of international research institutes of its kind.” The Institute has made solid strides towards its goal of developing into a world-class scientific research institute.

The glorious 60-year history of the Institute of Biophysics has been built upon the teamwork, perseverance, and efforts of several generations, led by senior scientists such as Shizhang Bei, Chenlu Tsou, Dong-cai Liang, and Fuyu Yang. Over the past 60 years, the staff of the Institute have explored the mysteries of life and served the needs of the nation with conscientious scholarship, diligence, and innovation. Thanks to their efforts, the Institute continues to thrive and grow, validating a basic principle of the Institute—talents are its foundation. More than ever, the Institute needs to attract a team of world-class leading experts, young scientists, and advanced innovation teams to fulfill its mission and create a better future. The Institute has laid a solid foundation for achieving this goal. Our current staff team includes 12 academicians, 25 scientists selected to China’s “Thousand Talents Program” (including 16 Young Talents), 15 scientists selected to the “Ten-Thousand Talents Program” (including three Young Top Talents), and 38 scientists selected to the CAS “Hundred Talents Program.” Thirty-eight of our staff are members of the Youth Innovation Promotion Association of the CAS, 24 have awards from the “National Science Fund for Distinguished Young Scholars,” and 12 have awards from the “Outstanding Youth Science Foundation.” Four groups from the Institute have been recognized as “Innovation Groups,” and two as “Innovation Teams” in key areas of the “Innovative Talents Promotion Program” of China’s Ministry of Science and Technology. In 2011, the Institute was awarded the status of a National Innovation and Entrepreneurship Base for Overseas High-level Talents by the Organization Department of the Central Committee of the CPC, and in 2014, it was among the second batch of organizations to be given the status of National Demonstration Base for Innovative Talent Training by the Ministry of Science and Technology.

In the current new era, China’s leaders have called for the nation to build itself into a world-class science and technology power. The life sciences need to become more deeply integrated with other disciplines and technologies in order to generate original breakthroughs that will contribute to the prosperity of the country. The Institute will fully execute the “Pioneer Initiative” by focusing on cutting-edge frontiers in science and technology, the needs of the country, and the national economy. It will also utilize its multi-disciplinary strengths, and further strengthen the construction of talent teams, key technology innovation and equipment R&D, and its innovation capacity. In this way, it will achieve fundamental, prospective, and strategic breakthroughs in the frontiers of the life sciences and make a substantial scientific and technological contribution to the revival of the Chinese nation, the wellbeing of the Chinese people, and the prosperity of the world.

On the 60th anniversary of the founding of the Institute of Biophysics, *Biophysics Reports* has published this commemorative special issue, inviting a number of outstanding scholars to write reviews and research articles to demonstrate the Institute’s academic research achievements in recent years and its current level of academic research. The publication of this issue aims to further enhance cooperation and communication between the Institute and academic colleagues in the field.

In light of the publication of this special issue, I would like to take this opportunity on behalf of the scientific and technical staff and students of the Institute, to express my heartfelt gratitude to both domestic and international peers and experts, friends, and people from all walks of life who have shown a long-term regard and support for the establishment and development of the Institute of Biophysics.


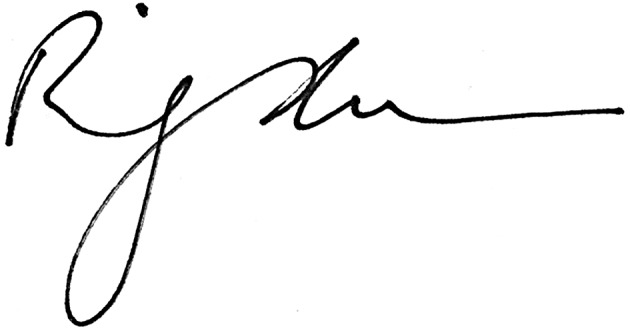


Rui-Ming Xu

Director, Institute of Biophysics, CAS

